# Exosome-Derived LINC00960 and LINC02470 Promote the Epithelial-Mesenchymal Transition and Aggressiveness of Bladder Cancer Cells

**DOI:** 10.3390/cells9061419

**Published:** 2020-06-07

**Authors:** Cheng-Shuo Huang, Jar-Yi Ho, Jung-Hwa Chiang, Cheng-Ping Yu, Dah-Shyong Yu

**Affiliations:** 1 Graduate Institute of Pathology and Parasitology, National Defense Medical Center, Taipei 114, Taiwan; qo4m4443151@gmail.com (C.-S.H.); jaryiho@gmail.com (J.-Y.H.); 2 Graduate Institute of Life Sciences, National Defense Medical Center, Taipei 114, Taiwan; 3 Department of Pathology, Catholic Cardinal Tien Hospital, Taipei 231, Taiwan; qo4m4139339@gmail.com; 4 Department of Pathology, Tri-Service General Hospital, National Defense Medical Center, Taipei 114, Taiwan; 5 Division of Urology, Department of Surgery, Tri-Service General Hospital, National Defense Medical Center, Taipei 114, Taiwan

**Keywords:** bladder cancer, exosome, epithelial-mesenchymal transition, long non-coding RNAs

## Abstract

Exosomes are essential for several tumor progression-related processes, including the epithelial–mesenchymal transition (EMT). Long non-coding RNAs (lncRNAs) comprise a major group of exosomal components and regulate the neoplastic development of several cancer types; however, the progressive role of exosomal lncRNAs in bladder cancer have rarely been addressed. In this study, we identified two potential aggressiveness-promoting exosomal lncRNAs, LINC00960 and LINC02470. Exosomes derived from high-grade bladder cancer cells enhanced the viability, migration, invasion and clonogenicity of recipient low-grade bladder cancer cells and activated major EMT-upstream signaling pathways, including β-catenin signaling, Notch signaling, and Smad2/3 signaling pathways. Nevertheless, LINC00960 and LINC02470 were expressed at significantly higher levels in T24 and J82 cells and their secreted exosomes than in TSGH-8301 cells. Moreover, exosomes derived from LINC00960 knockdown or LINC02470 knockdown T24 cells significantly attenuated the ability of exosomes to promote cell aggressiveness and activate EMT-related signaling pathways in recipient TSGH-8301 cells. Our findings indicate that exosome-derived LINC00960 and LINC02470 from high-grade bladder cancer cells promote the malignant behaviors of recipient low-grade bladder cancer cells and induce EMT by upregulating β-catenin signaling, Notch signaling, and Smad2/3 signaling. Both lncRNAs may serve as potential liquid biomarkers for the prognostic surveillance of bladder cancer progression.

## 1. Introduction

Bladder cancer is the seventh most prevalent cancer worldwide and the second most common urological malignancy behind prostate cancer. Its incidence is rising in most countries, with an estimated 549,393 new cases diagnosed in 2018 and 990,724 new cases expected in 2040 [1,2]. In Taiwan, the incidence of bladder cancer is also increasing annually [3]. Bladder cancer exhibits a high frequency of relapse and a poor clinical outcome once the tumors progress to muscle invasion and drug resistance [4,5,6,7,8]. Therefore, it is imperative to clarify the mechanisms that underlie the progression of bladder cancer aggressiveness from low-grade to high-grade bladder cancer and the frequent progression to muscle-invasive bladder cancer.

During epithelial to mesenchymal transition (EMT), epithelial cells are converted to migratory and invasive cells [9]. Loss of *E*-cadherin expression has long been considered to be a leading feature of EMT that leads to the loss of the typical polygonal, cobblestone morphology of epithelial cells. Alternatively, the cells acquire a spindle-shaped mesenchymal morphology and express markers that are associated with the mesenchymal cell state including neural cadherin (*N*-cadherin), vimentin and fibronectin [10]. The EMT program is triggered by the activation of several core transcription factors, including Snail, Slug, Zeb1/2, and Twist [11]. Recently, several reports have found that long non-coding RNAs (lncRNAs) play a regulatory role in tumor invasion and metastasis through EMT-based mechanisms in bladder cancer [12,13]. LncRNAs are no protein-coding transcripts with more than 200 nucleotides [14], which participate in a variety of physiological and pathological processes, especially in cancer initiation or progression [15,16].

Exosomes are membrane-derived vesicles containing proteins, double-stranded DNA, mRNA, miRNA, and lncRNA and participate in a variety of intercellular transmissions [17,18]. Exosomes also promote the EMT program and may thereby play a crucial role in delivering oncogenic factors to regulate cell proliferation, motility and stress tolerance by functioning as intracellular mediators taken up by recipient cells [19,20,21]. Although many lncRNAs, such as LINC00152, LINC00355, LINC00958, and urothelial carcinoma-associated 1 (UCA1), have been reported to be closely related to the pathogenesis, progression, and chemoresistance of bladder cancer [22,23,24,25], the regulatory role of exosomal lncRNAs in bladder cancer has rarely been addressed even though lncRNAs comprise a major group of exosomal components. Nowadays, there are three studies available on the PubMed that elucidate the regulatory role of exosomal lncRNA in bladder cancer. These are: exosomal lncRNA PTENP1 suppresses bladder cancer progression by competitively binding to oncomiR miR-17 and increases the expression of PTEN [26]; hypoxic exosomal lncRNA UCA1 promotes tumor growth and progression though EMT [27]; and exosomal lncRNA LNMAT2 promotes lymphatic metastasis of bladder cancer [28]. Here, we aimed to elucidate the intercellular communication of exosome-derived lncRNAs from high-grade bladder cancer cells to low-grade ones and their regulation of cancer progression and aggressiveness.

In this study, two potent candidate lncRNAs, LINC00960 and LINC02470, were screened out based on a series of selective criteria. After validation of their intracellular and exosomal expression levels, their biological effects and molecular regulation were evaluated, and the results indicated that exosome-derived LINC00960 and LINC02470 from high-grade bladder cancer cells aggravates malignant behaviors in low-grade bladder cancer cells and promotes the EMT process.

## 2. Materials and Methods

### 2.1. Cell Lines

Four human bladder cancer cell lines (low-grade, TSGH-8301 and TSGH-9202, and high-grade, T24 and J82) were originally acquired from American Type Cell Collection or the Bioresource Collection and Research Center for use in this study. All cells were incubated in RPMI 1640 medium containing 10% fetal bovine serum, 1 μg/mL penicillin and 1 μg/mL streptomycin (Life Sciences, Palo Alto, CA, USA) at 37 °C in a 5% CO_2_ humidified incubator.

### 2.2. Conditioned Medium Collection and Isolation of Exosomes from Conditioned Media

**Figure 1:**
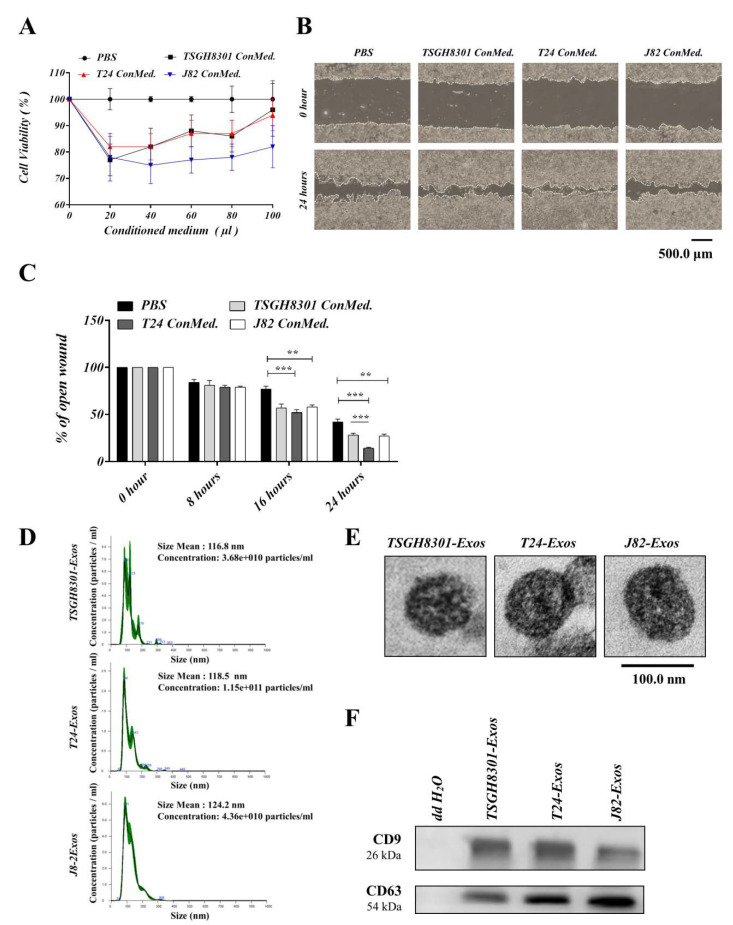
Conditioned medium of high-grade bladder cancer cells increased viability and motility of low-grade bladder cancer cells. (**A**) Cell viability was compared using MTT assays in TSGH-8301 cells treated with the indicated conditioned medium, ** *p* < 0.01, *** *p* < 0.001. (**B**,**C**) The wound healing assay demonstrated that conditioned medium increased the migration of TSGH-8301 cells. Wound areas were measured at 0, 8, 16, and 24 h after scratching, and the representative images were shown at 0 h and 24 h after scratching. The wound closure distance was measured with the ImageJ software. The bars represent the mean and SD of three independent experiments, ** *p* < 0.01, *** *p* < 0.001. (**D**) Nanoparticle tracking analysis was used to compare the average size of isolated exosomes. (**E**) Transmission electron microcopy was used to observe the morphology of isolated exosome. (**F**) Western blots revealed the presence of exosomal markers, CD9 and CD63, in isolated exosomes.

Conditioned media (ConMed) was derived from TSGH-8301, T24 or J82 cells. In brief, 5 × 10^6^ cells were seeded in a T75 flask and maintained in 10 mL RPMI-1640 containing 10% exosome-depleted FBS (SBI System Biosciences, Palo Alto, CA, USA), 1 μg/mL penicillin and 1 μg/mL streptomycin (Life Sciences) for 48 h, and whole cultured medium was collected and centrifuged in 3000× *g* for 10 min to remove cell debris. The supernatant was collected as conditioned medium for treated TSGH-8301 cells to evaluate their effects on cell viability and motility. Different volumes of condition medium (as indicated in Figure 1A) was mixed as above with complete RPMI-1640 medium to obtain 200 μL per well for evaluating cell viability with MTT assay. Conditioned medium and fresh complete medium with a 1:1 ratio was used for evaluating wound-healing assay.

Exosomes were isolated by differential centrifugation of conditioned media collected from TSGH-8301, T24 and J82 cells. Cells were grown in medium containing 10% exosome-depleted FBS (SBI System Biosciences, Palo Alto, CA, USA). After removing cells and other debris by centrifugation at 3000× *g* for 30 min, the supernatant was subsequently centrifuged at 10,000× *g* for 1 h to remove shedding vesicles and other large vesicles. Finally, the supernatant was recentrifuged at 120,000× *g* for 3 h at 4 °C. The exosome pellets were resuspended in PBS and stored at 4 °C before experimental analyses.

### 2.3. Nanoparticle Tracking Analysis

The number and size of exosomes were directly tracked using the NanoSight NS 300 system (NanoSight Technology, Malvern, UK). Exosomes were resuspended in PBS at a concentration of 5 μg/mL and further diluted 100-fold to achieve a concentration between 20 and 100 objects per frame. Samples were manually injected into the sample chamber at ambient temperature. Each sample was detected in triplicate with a 488-nm laser and a high-sensitivity scientific complementary metal-oxide semiconductor camera at a camera setting of 13 with an acquisition time of 60 s and a detection threshold setting of 7. The detection threshold was similar in all the samples and was applied using NTA 3.0 analytical software.

### 2.4. Transmission Electron Microscopy

For conventional transmission electron microscopy, the exosome pellet was placed in a droplet of mixed buffer 1:1 of 2.5% glutaraldehyde (in 0.1 M sodium cacodylate, pH 7.4) and 4% paraformaldehyde (in 1× PBS)) and fixed overnight at 4 °C. Samples were rinsed in PBS buffer (3 times, 10 min each) and further fixed in 1% osmium tetroxide (in double distilled water) for 50 min at room temperature. The samples were then embedded in 10% gelatin, fixed in glutaraldehyde at 4 °C, and cut into tiny blocks (<1  mm^3^). The samples were dehydrated with an alcohol gradient (70%, 90%, 95%, and 100%) for 10 min at each step. Pure alcohol was then exchanged with propylene oxide, and specimens were embedded in increasing concentrations (25%, 50%, 75% and 100%) of Quetol-812 epoxy resin mixed with propylene oxide for a minimum of 2 h per step. Samples were embedded in pure, fresh Quetol-812 epoxy resin and polymerized at 70 °C for 24 h. Ultrathin sections (300 nm) were cut using a Leica UC6 ultramicrotome. After staining with uranyl acetate for 10 min and lead citrate for 5 min at room temperature, exosome morphology was observed by transmission electron microscopy (Hitachi, HT7700, Japan) under operation at 120 kV and adjusted to the appropriate zoom.

### 2.5. Dynamic Uptake of Exosomes

Isolated exosomes were labeled with FITC-conjugated CD9 antibody (CD9-FITC-labeled exosomes). TSGH-8301 cells were pretreated with Hoechst DNA counterstain (Sigma-Aldrich, San Jose, CA, USA) for 24 h. Subsequently, cells were treated with CD9-FITC-labeled exosomes in serum-free medium. Treated cells were photographed for their entirety using the phase-contrast status at low power assessment every 30 min, and fluorescent images were photographed spontaneously with the Hoechst DNA channel (360 nm/460 nm excitation/emission) and FITC channel (400 nm/455 nm excitation/emission) using a Lionheart FX Automated Microscope. The dynamic uptake of exosomes was recorded using a BioTek Lionheart FX microscope (BioTek, Winooski, VT, USA). All experiments were performed in triplicate.

### 2.6. Cell Viability Assay

The viability of the bladder cancer cells was determined using the 3-[4,5-dimethylthiazol-2-yl]-2,5-diphenyl-tetrazolium bromide (MTT, Sigma-Aldrich) assay. Cells were seeded in 96-well plates at 5000 cells/well and cultured overnight. After treatment with the indicated concentrations of conditioned media or exosomes for 48 h, cells were incubated with 0.1 mg/mL MTT for 3 h, and formazan was dissolved in dimethyl sulfoxide (Sigma-Aldrich) at room temperature for 10 min. The absorbance at 560 nm was measured with a spectrophotometer (Bio-Rad Inc., Hercules, CA, USA). All experiments were performed in triplicate.

### 2.7. Cell Migration and Invasion Assay

In wound healing assays, 1 × 10^5^ cells were seeded in 6-well plates and incubated to 90% confluence before transfection. After treatment with the indicated concentrations of conditioned media or exosomes for 24 h, cells were scraped with a sterile 200 μL pipette tip to generate a clear line in the wells at time 0. The migrated cells were observed with a phase-contrast microscope every 8 h (Leica DMI4000B, Bucks, UK), and the wound width at the designated time was measured with ImageJ software. All experiments were performed in triplicate.

Transwell migration assays were performed using 8 μm transwell chambers (Corning, Steuben County, NY, USA) with 1 × 10^4^ cells for each exosome treatment. Transwell invasion assays were evaluated with the same chambers-coated with 1 mg/mL Matrigel (BD Biosciences, Franklin Lakes, NJ, USA) with 2 × 10^4^ cells for each exosome treatment. The migration and invasion chambers were incubated in a humidified 5% CO_2_ incubator at 37 °C for 24 h. Cells were then fixed with 4% paraformaldehyde, and the inner surface of the upper chambers was wiped with cotton swabs to remove unmigrated or uninvaded cells. After washing, the chambers were stained with crystal violet (Sigma-Aldrich) for 15 min, and the transwell membranes were torn and kept in slides. Five random fields of each treatment were photographed at 100× magnification, and the crystal violet-stained area was calculated using ImageJ software. Each condition was plated in triplicate.

### 2.8. Colony Formation Assays

The effects of exosomes on clonogenicity were evaluated with a colony-formation assay. In brief, 0.5 mL of 0.5% agarose in complete medium was used as the bottom agar in a 12-well plate, and 2 × 10^4^ cells were mixed with 0.3% agarose in complete medium at 48 h after treatment with the indicated concentrations of exosomes. Cells were maintained in a humidified 5% CO_2_ incubator at 37 °C for 21 days with fresh medium replacement every three days. Cell colonies were stained with crystal violet (Sigma-Aldrich) for 1 min and destained with tap water for 15 min. Colonies were counted using ImageJ software for each well, and triplicate repeats were performed for each condition.

### 2.9. Western Blotting

Total protein was extracted from cultured cells and exosomes using RIPA lysis and extraction buffer (Thermo Fisher Scientific, Waltham, MA, USA) supplemented with a protease inhibitor cocktail (Roche, Basel, Switzerland) at a ratio of 100:1. The protein concentration was determined by a BCA protein assay kit (Thermo Fisher Scientific, Waltham, USA). Thirty micrograms of protein was subjected to 10% SDS-PAGE and then transferred onto a polyvinylidene fluoride (PVDF) membrane (Millipore, Burlington, MA, USA). The membrane was blocked with 5% nonfat milk in TBST buffer and incubated with primary antibodies against CD9, CD63, beta-catenin, TCF4, Notch1, Notch4, HES1, Smad2/3, p-Smad2/3, Slug, Snail, Twist, Zeb2, *E*-cadherin, *N*-cadherin, vimentin, MMP2, MMP9, α-SMA, fibronectin or GAPDH (Abcam, Cambridge, UK and Cell Signaling Technology, Danvers, MA, USA) overnight at 4 °C. Subsequently, the blots were washed with TBST, followed by incubation with HRP-conjugated goat anti-mouse or goat anti-rabbit secondary antibody (1:5000; Santa Cruz Biotechnology, Dallas, TX, USA) at room temperature for 1 h. The immunoreactive bands were visualized with Immobilon Western Chemiluminescent HRP Substrate (Millipore, Burlington, MA, USA) and analyzed with the UVP GelStudio PLUS System (Analytik Jena AG, Thuringia, Germany). Each condition was performed in triplicate.

### 2.10. RT-qPCR Assay

Total RNA was extracted from cultured cells and exosomes using TOOLSmart RNA Extractor (BIOTOOLS, Taiwan (R.O.C.)). The concentration of total RNA was evaluated using a NanoDrop spectrophotometer (Thermo Fisher Scientific). Total RNA was reverse transcribed into cDNA using the ToolsQuant II Fast RT Kit with Oligo (dT) primer (BIOTOOLS) in a 20 μL reaction system consisting of 1000 ng template, 2 μL of 10× RT Reaction Premix with Oligo (dT) primer, 1.5 μL of ToolsQuant II Fast RT and RNase-free ddH_2_O. The mixture was centrifuged briefly and incubated with reverse transcription at 42 °C for 15 min, followed by enzyme inactivation at 85 °C for 5 min. Quantitative real-time PCR was performed in a 20 μL reaction system containing 3 μL of diluted cDNA, 10 μL of TOOLS 2X SYBR qPCR Mix (BIOTOOLS), 0.5 μL of gene-specific forward and reverse primers (10 pmol/μL) and 6.5 μL RNase-free ddH_2_O on a QuantStudio 5 Real-Time PCR System (Applied Biosystems, Foster City, CA, USA) according to the manufacturer’s instructions. Supplementary Materials, Table S1 lists the primer sequences used in this study. Briefly, after an initial denaturation step at 95 °C for 15 min, amplifications were carried out with 45 cycles at a melting temperature of 95 °C for 15 s, an annealing temperature of 60 °C for 20 s and elongation temperature of 72 °C for 20 s. The specificity of amplicons was confirmed by melting curve analysis. GAPDH was used as a reference gene. The relative expression levels of target genes were calculated using the 2^−ΔΔCT^ method. All experiments were conducted in triplicate, and no-template controls were included in each run.

### 2.11. Knockdown of LINC00960 and LINC02470

The LINC00960 and LINC02470 siRNAs and their negative control (NC) siRNAs were purchased from Dharmacon (GE Healthcare Dharmacon, Lafayette, CO, USA). T24 cells were transfected with 40 nM siRNA oligos or NC using Lipofectamine 2000 reagents (Invitrogen) according to the manufacturer’s instructions. The cells were harvested for RNA extraction, protein extraction or functional assays after 48 h of transfection of each siRNA, and RT-qPCR was used to determine the transfection efficiency. The data were analyzed with CELL Quest software. Each experiment was conducted in triplicate.

### 2.12. Statistical Analysis

All statistical analyses were performed with SPSS 17.0 (IBM, SPSS, Chicago, IL, USA) and GraphPad Prism 8.0 (GraphPad Software, La Jolla, CA, USA). Data were recorded as continuous variants and analyzed with Student’s *t* test. The results are expressed as the mean ± standard deviation of at least three separate experiments. All statistical tests and *p* values were two-sided, and the level of significance was set to < 0.05 (*), < 0.01 (**) or < 0.001 (***).

## 3. Results

### 3.1. Conditioned Media of High-Grade Bladder Cancer Cells Increased the Cell Viability and Motility of Recipient Low-Grade Bladder Cancer Cells

To evaluate the regulatory effects of ConMed from different cells, cell viability and motility were compared after three days of treatment with each ConMed. Four groups were treated with TSGH-8301-ConMed, T24-ConMed, or J82-ConMed in a series of volumes (0, 20, 40, 60, 80, and 100 μL), and the PBS control served as the comparison baseline. TSGH-8301 cells treated with any ConMed had a slower growth rate than that of the PBS control, which implied that each ConMed contained debris and metabolites and decreased the cell growth rate. However, each ConMed markedly increased the growth rate in a dose-dependent manner when a dose of more than 40 μL ConMed was added (Figure 1A). Moreover, compared to the PBS control, T24-ConMed increased the migratory ability in the wound healing assay (Figure 1B,C). These results suggest that some component(s) in the conditioned media of higher-grade bladder cancer cells possess the ability to promote the migration of TSGH-8301 cells and that exosomes transmission should be competent for intercellular communication.

### 3.2. Qualitative and Quantitative Detection of Exosomes

Bladder cancer cell-derived exosomes were isolated by the ultracentrifuge method from the conditioned media of TSGH-8301, T24 and J82 cells. NTA and TEM showed that the mean size and concentration of exosomes (Exos) were as follows: TSGH8301-Exos: mean size 116.8 ± 3.0 nm and concentration 3.68 × 10^10^ particles/mL; T24-Exos: mean size 118.5 ± 0.8 nm and concentration 1.15 × 10^11^ particles/mL; and J82-Exos: mean size 124.2 ± 1.0 nm and concentration 4.36 × 10^10^ particles/mL (Figure 1D,E). Western blotting revealed that the exosomal surface markers, CD9 and CD63 [29] were present on exosomes derived from each cell line (Figure 1F).

### 3.3. Exosomes Serve as Mediators of Intercellular Communication

**Figure 2:**
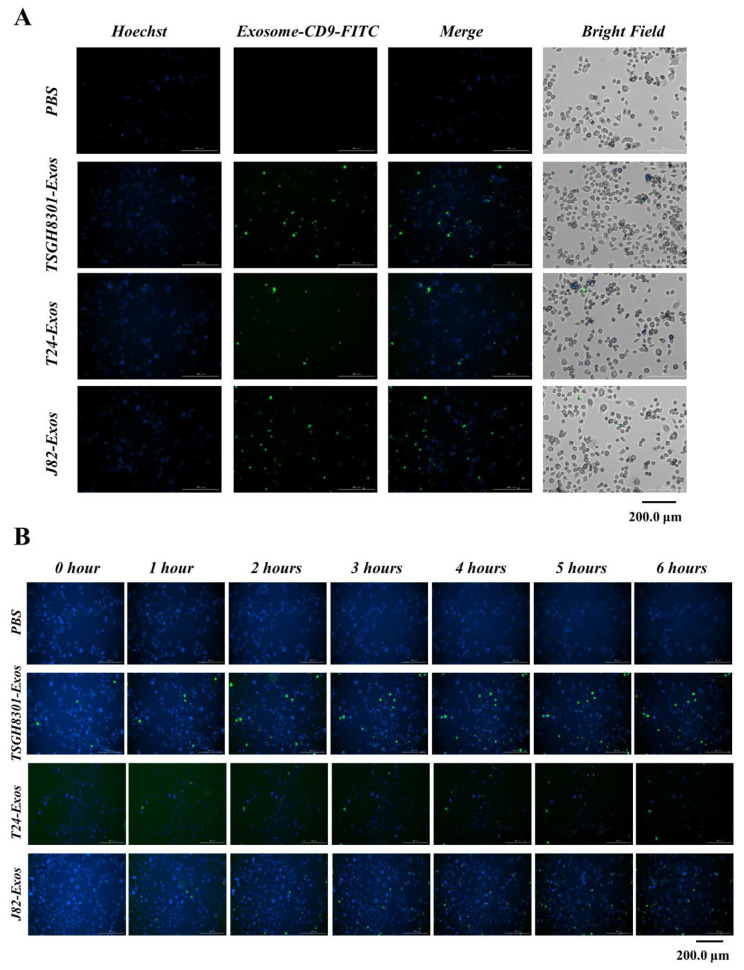
Exosomes serve as a mediator in intercellular communication. Exosomes derived from bladder cancer cells were labeled with FITC-conjugated anti-CD9 antibody (green) and counterstained with Hoechst 33342 (blue) for 24 h, and exosomes uptake was observed under a BioTek LIONheart FX microscope (**A**) for different spectra, and (**B**) merged figures were aligned upon different time points.

To evaluate the exosomes’ ability to uptake recipient TSGH-8301 cells, TSGH-8301 cells were treated with CD9-FITC-labeled exosomes then recorded every 30 min under the Hoechst DNA counterstain background. The staining intensity of the CD9-FITC-labeled exosomes was markedly increased (Figure 2A) in a time-dependent manner; staining tended to become saturated before 6 h (Figure 2B). These results indicated that the exosome uptake rates in TSGH-8301 cells were similar among those exosomes derived from different cells (PBS: Video S1; TSGH8301-Exos: Video S2; T24-Exos: Video S3; J82-Exos: Video S4).

### 3.4. T24-Exos and J82-Exos Promote the Malignant Behavior of Recipient TSGH-8301 Cells

**Figure 3:**
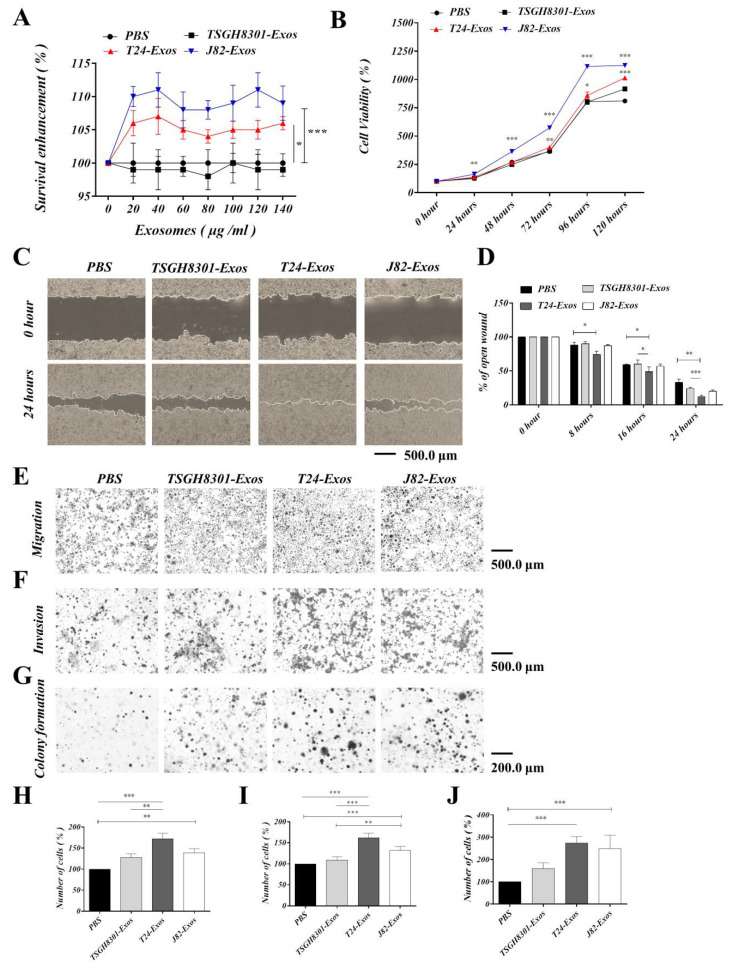
T24-Exos and J82-Exos intensified the aggressive behavior of TSGH-8301 cells. (**A**) Cell viability of different concentrations and (**B**) cell growth curve of different time points were compared using MTT assays in TSGH-8301 cells treated with each exosome, * *p* < 0.05, ** *p* < 0.01, *** *p* < 0.001. (**C**,**D**) The wound healing assay demonstrated that T24 and J82 cell-derived exosomes increased the migratory abilities of TSGH-8301 cells. Wound areas were photographed at 0, 8, 16, and 24 h after scratching, and representative images are shown at 0 h and 24 h after scratching. The wound closure distance was calculated with the ImageJ software. The bars represent the mean and SD of three independent experiments, * *p* < 0.05, ** *p* < 0.01, *** *p* < 0.001. (**E**,**H**) Transwell assay showed that T24 and J82 cell-derived exosomes increased the migratory abilities of TSGH-8301 cells. (**F**,**I**) Matrigel-coated transwell assay showed that T24 and J82 cells derived exosomes increased the invasive abilities of TSGH-8301 cells. Representative images of the (**E**) migrated and (**F**) invaded cells were photographed at 24 and 72 h after cell inoculation, respectively (**G**,**J**) Soft-agar colony formation assay was performed to examine the anchorage-independent survival of cells. The bar-charts showed the quantitative change in (**H**) migration, (**I**) invasion, and (**J**) cell clones, respectively. All experiments were performed at least in triplicate, * *p* < 0.05, ** *p* < 0.01, *** *p* < 0.001.

To evaluate the biological effects of T24-Exos and J82-Exos on regulating cell viability, migration and invasion of TSGH-8301 cells, recipient TSGH-8301 cells were treated with increasing concentrations of TSGH-8301-Exos, T24-Exos, or J82-Exos (0, 20, 40, 60, 80, 100, 120 and 140 μg/mL) and PBS control for 48 h. Compared to the control, T24-Exos and J82-Exos significantly increased the cell viability of TSGH-8301 cells in a concentration-dependent manner, but the inductive effect reached a plateau pattern when the concentration of exosomes was higher than 40 μg/mL (Figure 3A). Then, the time-dependent effects on cell viability were evaluated with 40 μg/mL of each exosome. T24-Exos and J82-Exos significantly promoted the viability of TSGH-8301 cells, especially J82-Exos, which demonstrated more potent promotive effects after 24 h of treatment (Figure 3B). Furthermore, T24-Exos and J82-Exos also significantly promoted the migratory (Figure 3C–E,H), invasive (Figure 3F,I), and clonogenicity (Figure 3G,J) abilities of TSGH-8301 cells. Thus, after exposure to T24-Exos and J82-Exos, increased cell viability and motility were observed in recipient TSGH-8301 cells in dose- and time-dependent patterns.

### 3.5. The Expression of Epithelial–Mesenchymal Transition-Associated Molecules Was Promoted after Exosome Treatment

**Figure 4:**
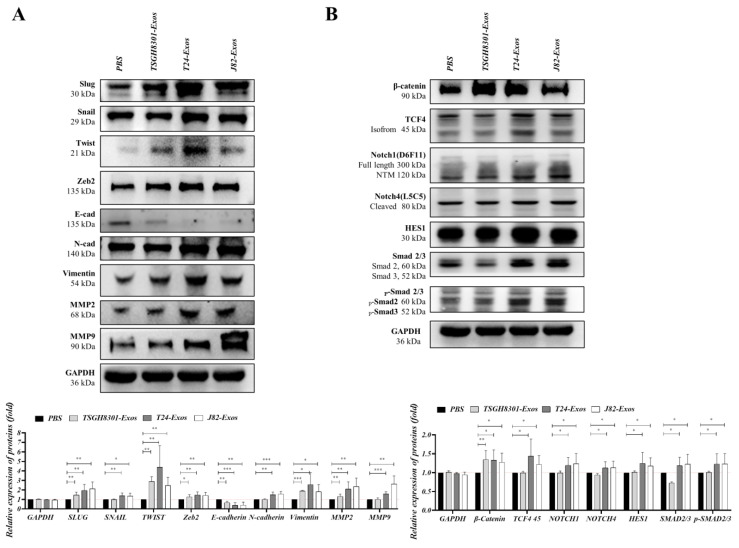
Exosomes mediated the expression of epithelial–mesenchymal transition-related molecules. (**A**) Protein expression levels of EMT effectors: Slug, Snail, Twist, Zeb2, *E*-Cadherin, *N*-Cadherin, vimentin, MMP2, and MMP9, and (**B**) EMT-induced signaling molecules: β-catenin, TCF4, Notch1, Notch4, HES1, Smad2/3, and p-Smad2/3 were compared after treatment with exosomes. The bar-charts showed the mean and SD of triplicate experiments, * *p* < 0.05, ** *p* < 0.01, *** *p* < 0.001.

T24-Exos and J82-Exos had higher inductive effects on cell motility than cell viability, which indicates that T24-Exos and J82-Exos participate in some signaling pathways that significantly induce cell migration and moderately increase cell proliferation. Therefore, the EMT process seemed to be a potential candidate for further analyses. TSGH-8301 cells were separately treated with PBS control, TSGH-8301-Exos (40 μg/mL), T24-Exos (40 μg/mL), and J82-Exos (40 μg/mL) for 3 days, and treated cells were harvested for Western blotting. In Figure 4A, EMT transcription factors (EMT-TF) were first compared, and Snail, Slug and Zeb2 were significantly upregulated after T24-Exos or J82-Exos treatment, while Twist was significantly upregulated after T24-Exos treatment compared to PBS control treatment. Subsequently, markers of epithelial-like or mesenchymal-like properties were compared, and the epithelial marker *E*-cadherin was significantly reduced after T24-Exos or J82-Exos treatment, although it was also slightly reduced after TSGH-8301-Exos treatment. Conversely, the mesenchymal marker *N*-cadherin, α-SMA, fibronectin (Figure S2A) and the interstitial indicator vimentin were increased after T24-Exos or J82-Exos treatment. Moreover, matrix metallopeptidases were analyzed to confirm the invasive potential and MMP2 and MMP9 expression levels were significantly increased after T24-Exos or J82-Exos treatment. In short, T24-Exos or J82-Exos induced EMT in recipient TSGH-8301 cells by upregulating most EMT-TFs and resulted in a decrease in *E*-cadherin but an increase in *N*-cadherin, vimentin, MMP2 and MMP9. Second, three major upstream signaling cascades of the EMT process, including β-catenin/TCF4 signaling, Notch/HES1 signaling and TGF-β/SMAD signaling were analyzed. As shown in Figure 4B, β-catenin was significantly upregulated after TSGH8301-Exos, T24-Exos or J82-Exos treatment compared to the PBS control group. However, TCF4 was upregulated after T24-Exos treatment but was not affected by J82-Exos or TSGH8301-Exos treatment. T24-Exos or J82-Exos also induced Notch1, Notch4, and HES1 upregulation when compared to the PBS control group. In addition, T24-Exos or J82-Exos also induced the upregulation and phosphorylation/activation of Smad2/3. In contrast, TSGH-8301-Exos downregulated Smad2/3 and decreased their phosphorylation/activation. In summary, both T24-Exos or J82-Exos induced EMT in recipient TSGH-8301 cells, and three major upstream signaling cascades were also obviously activated after T24-Exos or J82-Exos treatment. These results suggested that there were some broad-spectrum regulators in the exosomes derived from high-grade bladder cancer cells, which led to the comprehensive induction of the EMT process in recipient TSGH-8301 cells.

### 3.6. Screening and Validation of Exosomal lncRNAs

**Figure 5:**
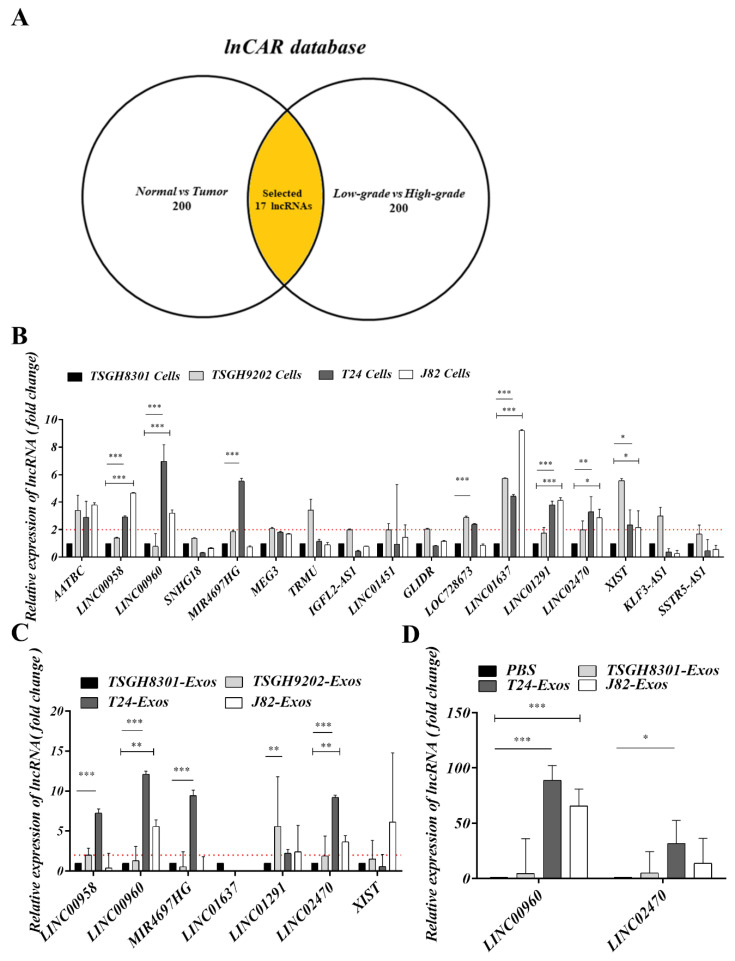
Screening and validation of lncRNA candidates. (**A**) Schematic diagram of lncRNA screening. Seventeen candidate lncRNAs were screened from the intersection between the top 200 lncRNA of normal vs. tumor and low-grade vs. high-grade bladder cancer datasets in the lnCAR database. (**B**) Intracellular expression levels of AATBC, LINC00958, LINC00960, SNHG18, MIR4697HG, MEG3, TRMU, IGFL2-AS1, LINC01451, GLIDR, LOC728673, LINC01637, LINC01291, LINC02740, XIST, KLF3-AS1, SSTR5-AS1 were among the different bladder cancer cells that were compared using RT-qPCR. (**C**) Exosomal levels of LINC00958, LINC00960, MIR4697HG, LINC01637, LINC01291, LINC02740 and XIST were also compared using RT-qPCR. (**D**) Exosome-derived LINC00960 or LINC02470 transferred into TSGH-8301 was also evaluated by RT-qPCR. The bar-charts showed the mean and SD of triplicate experiments, * *p* < 0.05, ** *p* < 0.01, *** *p* < 0.001.

LncRNAs comprise a considerable proportion of exosomal components, and several lncRNAs have been reported to contribute to multiple oncogenic steps, including tumor formation, progression, and/or metastatic processes in many cancer types; hence, this study was designed to evaluate the potential of exosome-derived lncRNAs to induce aggressiveness by promoting the EMT process. Exosome-derived lncRNA candidates were screened with bioinformatic pipelines from the lnCAR database (https://lncar.renlab.org/). In brief, the top 200 lncRNAs expressed more highly in tumoral than normal bladder tissues and the top 200 lncRNAs expressed more highly in high-grade bladder cancer than low-grade bladder cancer were selected. After overlapping those top differentially expressed lncRNAs (200 vs. 200), 17 potential candidates were intersected and subjected to RT-qPCR validation in bladder cancer cells and exosomes. A comparison of AATBC, LINC00958, LINC00960, SNHG18, MIR4697HG, MEG3, TRMU, IGFL2-AS1, LINC01451, GLIDR, LOC728673, LINC01637, LINC01291, LINC02740, XIST, KLF3-AS1, and SSTR5-AS1 expression was performed in TSGH-8301, TSGH-9202, T24 and J82 cells (Figure 5A,B). Seven lncRNAs, including LINC00958, LINC00960, MIR4697HG, LINC01637, LINC01291, LINC02740, and XIST, had expression levels that were at least two-fold higher in T24 and J82 cells than in TSGH-8301 cells. Their expression in exosomes was further compared. Only LINC00960 and LINC02470 were expressed in T24-Exos and J82-Exos at levels that were two-fold higher than those in TSGH-8301-Exos (Figure 5C). Therefore, subsequent analyses of the biological effects and molecular regulation of these two lncRNA candidates were performed. Intracellular LINC00960 and LINC02470 were significantly increased in recipient TSGH-8301 cells after T24-Exos or J82-Exos treatment, although the difference in LINC02470 expression did not reach statistical significance in the J82-Exos-treated group (Figure 5D).

### 3.7. Exosomes Derived from LINC00960 Knockdown or LINC02470 Knockdown T24 Cells Reduced Aggressive Behavior in Recipient TSGH-8301 Cells

**Figure 6:**
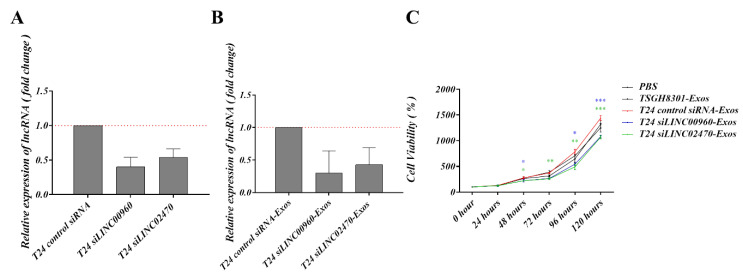
Exosomes derived from LINC00960-knockdown or LINC02470-knockdown T24 cell conferred less aggressiveness-inductive abilities in recipient TSGH-8301 cells. LINC00960 or LINC02470 expression levels were compared by RT-qPCR in (**A**) intracellular levels and (**B**) the exosomal levels. (**C**) Cell growth curve was compared using MTT assays in TSGH-8301 cell treated with each exosome. The quantitative results are shown as the bar-charts or growth curves for mean and SD of triplicate experiments, * *p* < 0.05, ** *p* < 0.01, *** *p* < 0.001.

**Figure 7:**
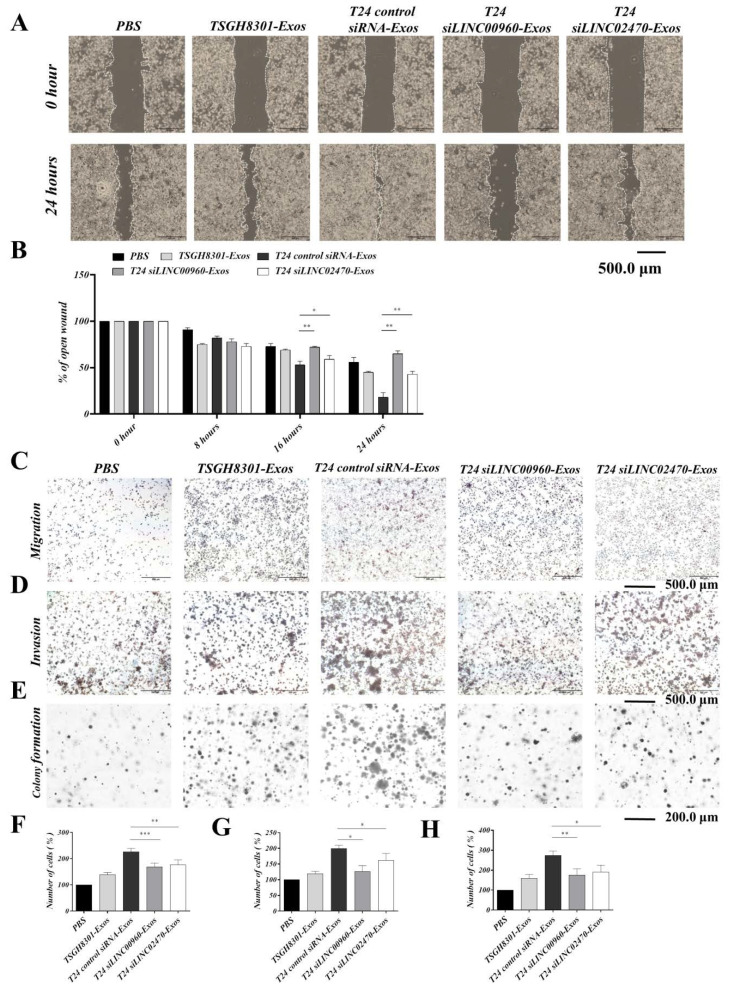
Exosomes derived from LINC00960-knockdown or LINC02470-knockdown T24 cell conferred less aggressiveness-inductive abilities in recipient TSGH-8301 cells. T24 siLINC00960-Exos or T24 siLINC02470-Exos induced less migratory ability (**A**,**B** for wound healing assay and **C**,**F** for transwell assay), invasive ability (**D**,**G** for Matrigel-coated transwell assay), and clonogenicity (**E**,**H** for soft-agar colony formation assay) of recipient TSGH-8301 cell when compared to T24-Exos. All experiments were performed with the identical time-points shown in Figure 3, and the bar-charts showed the mean and SD of triplicate experiments, * *p* < 0.05, ** *p* < 0.01, *** *p* < 0.001.

To verify the inhibitory efficacy of siRNA for LINC00960 (siLINC00960) or LINC02470 (siLINC02470), T24 cells were transfected with siLINC00960 or siLINC02470 and intracellular exosomal levels of these two lncRNAs were tested compared to siRNA control. As shown in Figure 6A, siLINC00960 specifically and significantly reduced LINC00960 expression by more than 50%, and siLINC02470 specifically and significantly reduced LINC02470 expression by ~50% at both the intracellular level (Figure 6A) and the exosomal level (Figure 6B) compared to that in the control siRNA groups. Moreover, the biological effects of exosomes derived from each siRNA transfectant were further assessed by detecting the cell viability, migration, invasion and clonogenicity of exosome-recipient TSGH-8301 cells. Compared to exosomes derived from control siRNA-transfected T24 cells, exosomes derived from siLINC00960-transfected T24 (T24 siLINC00960-Exos) or siLINC02470-transfected T24 (T24 siLINC02470-Exos) cells demonstrated significantly reduced inductive effects on cell viability (Figure 6C), cell migration (Figure 7A–C,F) and invasion (Figure 7D,G) clonogenicity (Figure 7E,H) Therefore, the aggressiveness-promoting abilities of T24-Exos in recipient TSGH-8301 cells were significantly attenuated after knockdown of either LINC00960 or LINC02470.

### 3.8. Exosomes Derived from LINC00960 Knockdown or LINC02470 Knockdown T24 Cells Reduced the Expression Levels of EMT-Associated Molecules in Recipient TSGH-8301 Cells

**Figure 8:**
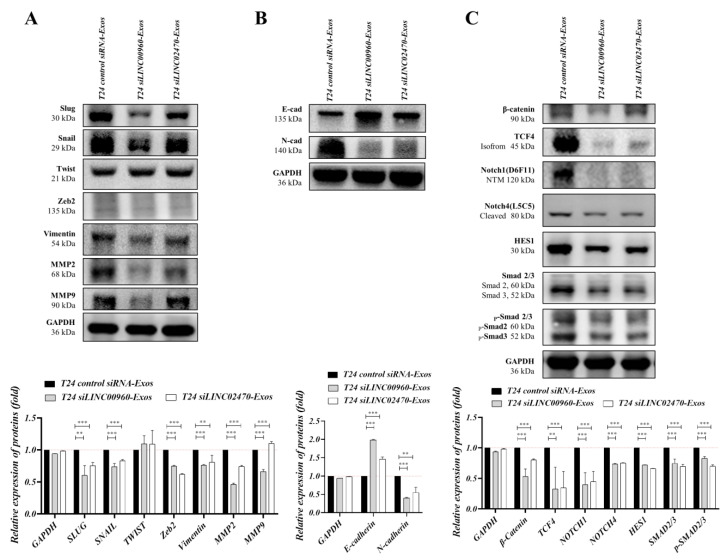
Exosomes derived from LINC00960-knockdown or LINC02470-knockdown T24 cell reduced the expression levels of EMT-related molecules in recipient TSGH-8301 cells. (**A**) Protein expression levels of EMT effectors: Snail, Slug, Twist, Zeb2, vimentin, MMP2, MMP9, (**B**) *E*-Cadherin and *N*-Cadherin, and (**C**) EMT-induced signaling molecules: β-catenin, TCF4, Notch1, Notch4, HES1, Smad2/3 and p-Smad2/3 were compared after treatment with each exosome. The bar-charts showed the mean and SD of triplicate experiments, ** *p* < 0.01, *** *p* < 0.001.

TSGH-8301 cells were separately treated with T24-Exos (40 μg/mL), T24 siLINC00960-Exos (40 μg/mL), and T24 siLINC02470-Exos (40 μg/mL) for 3 days, and treated cells were harvested for Western blotting. In Figure 8, EMT-TFs were compared, and Snail and Slug levels were significantly reduced after T24 siLINC00960-Exos or T24 siLINC02470-Exos treatment when compared to those in the T24-Exos-treated group. However, Twist and Zeb2 levels were similar among these three groups (Figure 8A). In addition, vimentin, MMP2 and MMP9 (Figure 8A), *N*-cadherin (Figure 8B), α-SMA and fibronectin (Figure S2B) levels were also significantly reduced after T24 siLINC00960-Exos or T24 siLINC02470-Exos treatment when compared to those in the T24-Exos-treated group, and *E*-cadherin levels were upregulated after T24 siLINC00960-Exos or T24 siLINC02470-Exos treatment (Figure 8B). Moreover, three major upstream signaling cascades of the EMT process described in Figure 4 were also analyzed. In Figure 8, T24 siLINC00960-Exos or T24 siLINC02470-Exos significantly reduced β-catenin and TCF4 expression (β-catenin signaling), Notch1, Notch4 and HES1 expression (Notch signaling), and Smad2/3 expression and their phosphorylation/activation (Smad2/3 signaling) when compared to that in the T24-Exos-treated group (Figure 8C). In summary, both T24 siLINC00960-Exos and T24 siLINC02470-Exos attenuated the T24-Exos-induced EMT process and its related regulatory mechanism in recipient TSGH-8301 cells. These results suggest that both LINC00960 and LINC02470 play comprehensive roles in EMT regulation and thereby promote the aggressiveness of recipient TSGH-8301 cells.

## 4. Discussion

Bladder cancer is usually multifocal with high recurrence and metastasis, and the EMT process has been shown to be comprehensively involved in bladder cancer progression [30,31]. Non muscle-invasive bladder cancer (NMIBC) accounts for more than 70% of newly diagnosed bladder cancers, and more than half of NMIBCs ultimately recur; approximately 15% of NMIBCs progress to muscle-invasive and/or metastatic diseases, which are highly connected with the EMT process [32,33]. The multifocality of bladder cancer has been hypothesized to originate from intraluminal monoclonal expansion (clonal) [34,35] or spontaneous transformation of multiple cells by virulent environmental agents (field effect) [36]. It is characterized by early genetic instability or aberrant epigenetic methylation [37,38,39]. Our findings indicate that exosomes function as potential environmental agents or the “field effect” to promote bladder cancer progression, which might lead to higher severity of multifocal tumors or more malignant behaviors of NMIBC cells.

The key regulators of the EMT process are *E*-cadherin, *N*-cadherin, vimentin, Snail, Slug, Twist, and Zeb-2, and most of these have been correlated with bladder cancer progression via either genetic or epigenetic regulation [40]. Currently, little is known about the association between the epigenetic regulation of exosome-derived lncRNAs in the EMT process and bladder cancer progression. In this study, we found that exosomes derived from high-grade bladder cancer cells did indeed promote the viability, motility, and clonogenicity of low-grade bladder cancer cells via enhancement of the EMT process. Furthermore, we identified two novel exosomal lncRNAs, LINC00960 and LINC02470, that both play pivotal roles in the EMT process and promote the aggressiveness of bladder cancer cells.

Exosomes are key elements that can facilitate intercellular communication and modulate tumor cells by influencing major cellular processes such as apoptosis, cell differentiation, angiogenesis and metastasis [41]. Bladder cancer cells have been reported to undergo EMT transformation after exposure to muscle-invasive bladder cancer exosomes [42]. Exosomes have also been reported to enhance cancer progression and recurrence in hepatocellular carcinoma via the MAPK/ERK signaling pathway [43]. Our results showed that low-grade bladder cancer cells (TSGH-8301) treated with high-grade bladder cancer cell-derived exosomes (T24-Exos or J82-Exos) had an increase in mesenchymal proteins (*N*-cadherin and vimentin) and a decrease in *E*-cadherin. T24-Exos or J82-Exos also significantly promoted the migratory and invasive abilities of recipient TSGH-8301 cells. The morphology of the recipient cells gradually changed into spindle-shaped mesenchymal-like cells after T24-Exos or J82-Exos treatment (Figure S1). In comparison, TSGH-8301-Exos treatment did not change their behaviors and morphology in an autocrine manner. This suggests that exosomes derived from cells of different aggressiveness lead to different levels of aggressiveness-promoting effects in recipient bladder cancer cells.

The three most representative EMT upstream pathways, β-catenin/TCF signaling, Notch signaling and Smad2/3 signaling were assessed to determine their involvement in exosome-induced EMT. Aberrant activation of β-catenin/TCF signaling is involved in a number of tumors, most notably colorectal carcinomas. TCF4 has been reported to transactivate Snail, Slug and Zeb1 and promote the EMT process [44,45]. Notch1 signaling increases the DNA binding ability of NF-κB and thereby induces the expression of MMP9, which remodels the extracellular matrix and facilitates the extravasation of several cancer cells. Notch also stabilizes cytoplasmic β-catenin and activates other pathways, such as ERK and NF-κB, which induce the expression of Snail, Slug and LEF-1 transcription factors [46,47,48]. Smad complexes bind to regulatory elements and induce the transcription of key genes associated with EMT. Expression of activated Smad2 promotes mesenchymal spindle tumor cell invasion, which also regulates the expression of Snail, Slug and Twist to suppress the expression of *E*-cadherin [49,50]. Our results showed that β-catenin expression can be induced by treatment with each type of exosome, but its downstream transcription factors, especially TCF4, were significantly induced by T24-Exo treatment alone. The expression levels of Notch1, Notch4, Smad2 or Smad3 were all significantly upregulated by T24-Exos or J82-Exos treatment. T24-Exos or J82-Exos also induced the expression of the EMT-transcription factors Slug, Snail, Twist and Zeb2, which ultimately suppressed *E*-cadherin expression but promoted *N*-cadherin, vimentin, MMP2 and MMP9 expression in bladder cancer cells.

Several lncRNAs are involved in the EMT process and the degree of malignancy of tumors [51]. The main advantage of lncRNAs is their high stability while circulating in body fluids, especially when they are encapsulated in exosomes or apoptotic bodies. This makes them suitable cancer diagnostic and prognostic biomarkers, [52]. Several lncRNAs are associated with bladder cancer initiation and progression. LINC00958 has a regulatory role in bladder cancer progression, as its knockdown decreases cell viability, migration, and invasion [24]. LINC00355 is upregulated in bladder cancer samples and contributes to apoptosis inhibition, cell proliferation, and migration [23]. The UCA1 lncRNA is also involved in cell proliferation, migration, invasiveness, and drug resistance of bladder cancer cells [25]. LINC00152 is highly expressed in bladder cancer and confers its carcinogenic effects by activating the Wnt/β-catenin signaling pathway [22]. Exosomes and their contained lncRNAs are regarded as components of cell signal transmission that modulate endogenous cellular microenvironments because exosomes are able to relocate functional lncRNAs between cells [53]. In this study, we identified two novel lncRNAs, LINC00960 and LINC02470, that were highly expressed in high-grade bladder cancer cells and their cell-derived exosomes. To our knowledge, this is the first study to address the function of LINC00960 and LINC02470, not only in intracellular regulation of malignancy traits but also in intercellular communication, which illustrates their powerful role in influencing the behaviors of bladder cancer cells. Our data demonstrated that siLINC00960-Exos or siLINC02470-Exos derived from T24 cells significantly inhibited proliferation, migration, invasion, and colony formation in recipient cells compared with their parental T24 exosomes. In addition, T24 siLINC00960-Exos treatment significantly reduced the migratory capability of recipient cells compared to siLINC02470-Exos treatment, which implies that LINC00960 and LINC02470 regulate the EMT process via different routes. Moreover, we found that T24 siLINC00960-Exos or siLINC02470-Exos treatment significantly reduced β-catenin signaling, Notch signaling and Smad2/3 signaling; notably, the major EMT transcription factors, Snail and Slug were significantly reduced, which led to the downregulation of *N*-cadherin and vimentin [54].

As mentioned above, some exosomal lncRNAs have been correlated with bladder cancer progression including exosomal PTENP1, which suppresses bladder cancer progression by sponging miR-17 and rescuing PTEN expression [26], exosomal UCA1 promotes tumor growth and progression under hypoxia [27], and exosomal LNMAT2 promotes lymphatic metastasis of bladder cancer [28]. Besides, several exosomal lncRNAs or lncRNAs panel have been examined in liquid biopsies of bladder cancer patients or relative controls to evaluate their diagnostic or prognostic value. For example, higher urinary exosomal LNMAT2 [28], higher urinary and serum exosomal circPRMT5, higher serum exosomal UCA1 [27], the PCAT-1, UBC1, SNHG16 panel [55], higher plasma exosomal H19 [56] and lower plasma exosomal PTENP1 were more frequently observed in bladder cancer patients than in healthy controls [26]. Additionally, higher urinary exosomal HOTAIR [57], MALAT1, PCAT-1 [58], higher plasma exosomal H19 [56], and higher serum exosomal UBC1 in NMIBC were associated with poorer prognosis of bladder cancer patients [55]. This should be clinically applicable in that higher urinary or serum exosomal LINC00960 and LINC02470 expression levels might be associated with higher pathological grade or poorer prognosis of bladder cancer patients. Moreover, high urinary exosomal LINC00960 and LINC02470 might indicate the high possibility of multifocality or local recurrence of bladder cancer.

**Figure 9:**
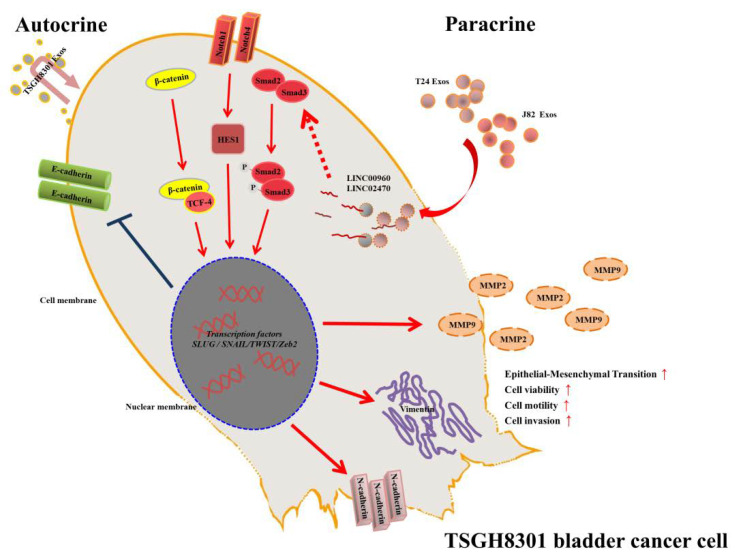
Schematic diagram of the LINC00960 and LINC02470-based signaling circuit in low-grade bladder cancer. T24-Exos and J82-Exos-derived LINC00960 and LINC02470 increased the expression of β-catenin signaling, Notch signaling, Smad2/3 signaling, transcription factors Snail, Slug, *N*-Cadherin, vimentin, MMP2, and MMP9, decreased *E*-Cadherin and promoted tumor progression of recipient TSGH-8301 cells.

In summary, we demonstrated that high-grade bladder cancer cell-derived exosomes promoted the malignant traits of low-grade bladder cancer cells. T24-Exos or J82-Exos-derived LINC00960 and LINC02470 induced the aggressiveness of bladder cancer cells via autocrine or paracrine effects. In contrast, T24 siLINC00960-Exos or siLINC02470-Exos attenuated these aggressiveness-promoting effects and inhibited the EMT process. Thus, T24-Exos or J82-Exos-derived LINC00960 and LINC02470 could be important aggressiveness-promoting factors in bladder cancer progression (Figure 9). It was also suggested that intercellular epigenetic regulations play critical roles during bladder cancer progression.

## 5. Conclusions

Our findings indicate that exosome-derived LINC00960 and LINC02470 derived from high-grade bladder cancer cells promote the aggressive behavior of low-grade bladder cancer cells through intercellular communication and promote EMT by promoting β-catenin signaling, Notch signaling and Smad2/3 signaling. Both lincRNAs may serve as potential liquid biomarkers for the prognostic surveillance of bladder cancer patients in the future.

## Supplementary Materials

The following are available online at https://www.mdpi.com/2073-4409/9/6/1419/s1, Figure S1, Figure S2, Table S1: Primer sequences in the study, Video S1: Exosome uptake under PBS treatment, Video S2: Exosome uptake under TSGH8301-Exos treatment, Video S3: Exosome uptake under T24-Exos treatment, Video S4: Exosome uptake under J82-Exos treatment.

## Abbreviations

ConMed	Conditioned medium
*E*-cad	*E*-cadherin
EMT	Epithelial-mesenchymal transition
Exos	Exosome
FBS	Fetal bovine serum
FITC	V-fluorescein isothiocyanate
GAPDH	Glyceraldehyde-3-phosphate dehydrogenase
HES1	Hes Family BHLH Transcription Factor 1
lncRNA	Long non-coding RNAs
lincRNA	Long intergenic non-coding RNA
*N*-cad	*N*-cadherin
NMIBC	Non muscle-invasive bladder cancer
NTA	Nanoparticle tracking analysis
MIBC	Muscle-invasive bladder cancer
MMPs	Matrix metalloproteinases
OD	Optical density
PBS	Phosphate buffer saline
RPMI	Roswell Park Memorial Institute
RT-qPCR	Reverse transcription quantitative polymerase chain reaction
TCF-4	Transcription factor 4
TSGH	Tri-Service General Hospital
Zeb2	Zinc finger E-box binding homeobox 2
