# Supplementary material for: Exosome-Derived LINC00960 and LINC02470 Promote the Epithelial-Mesenchymal Transition and Aggressiveness of Bladder Cancer Cells
Source: Cells. 2020 Jun 7;9(6):1419. doi: 10.3390/cells9061419 (PMC7349410; doi:10.3390/cells9061419)
Supplement: Supplementary file 1 [file cells-09-01419-s001.zip › cells-799613 suppl-final/Supplementary.pdf]

Supplementary Table S1. Primer sequences in the study

AATBC	Forward (5′-3′)	GGCTGGGCTCCAAAGCTTGA
	Reverse (5′-3′)	CTGTTGGTGTTGGCGTCCCT
LINC00958	Forward (5′-3′)	TGGATTCATGCTTCGCACCCC
	Reverse (5′-3′)	AGATCTCAGCAGCTCCGGCT
LINC00960	Forward (5′-3′)	CCTCTAAGCCTAAGCACCGCC
	Reverse (5′-3′)	GGAAGCCTGGGCAAGGAATGG
SNHG18	Forward (5′-3′)	CCCCACCTCACAGCCAAGTT
	Reverse (5′-3′)	TGCGAGTCAGCGCATCTTCT
MIR4697HG	Forward (5′-3′)	CCACGGTCTCTGCACAAGGG
	Reverse (5′-3′)	TGCATGTGGCCAGAAGTCGG
MEG3	Forward (5′-3′)	CTGAGGCCTAGGGGAGCTGT
	Reverse (5′-3′)	AGCCCTGTGCTTTGGAACCG
TRMU	Forward (5′-3′)	GGGGTCTGTACTGCCGACAA
	Reverse (5′-3′)	GCATCCTGGGAAACCTGGCATT
IGFL2-AS1	Forward (5′-3′)	GCCCCAAGGGCTCATTCACA
	Reverse (5′-3′)	TGTGTAAGTAACACGTCCTGGGT
LINC01451	Forward (5′-3′)	TGAGCTGTGCATCCCGTGTG
	Reverse (5′-3′)	CGCCACAGTCCGTGTGTGAT
GLIDR	Forward (5′-3′)	CGAATCCACCCATTGCCCGT
	Reverse (5′-3′)	GAGGCGTCTCTTCCGTGCAG
LOC728673	Forward (5′-3′)	TGACGGGCGAGGGTTACTGT
	Reverse (5′-3′)	CCGCTCAGGCTCAAGAGCAA
LINC01637	Forward (5′-3′)	ACCCTGAAAACGGGCTCGGA
	Reverse (5′-3′)	GCGATGCGGGGACATGAGTA
LINC01291	Forward (5′-3′)	GTGACCAGCAGCATGTGTTTCCA
	Reverse (5′-3′)	CAGGACTCACTGGCAATTCACCT
LINC02470	Forward (5′-3′)	CAGCAGACCAACACGCCAGA
	Reverse (5′-3′)	GCTCCATCTGCCCACATCCA
XIST	Forward (5′-3′)	TGGCCAACAGGTGGCAGAAG
	Reverse (5′-3′)	CCCCAGCATTAGCCAAGGGG
KLF3-AS1	Forward (5′-3′)	GATGAGCACACACACGTACGGA
	Reverse (5′-3′)	TCCACGCACTCATTCAGCCA
SSTR5-AS1	Forward (5′-3′)	GTGCTGCTGCCCTCGTCTAC
	Reverse (5′-3′)	CCTGTGCCTGGACTTCGCTC
GAPDH	Forward (5′-3′)	CCTGCCGGTGACTAACCCTG
	Reverse (5′-3′)	TCCACCACTGACACGTTGGC

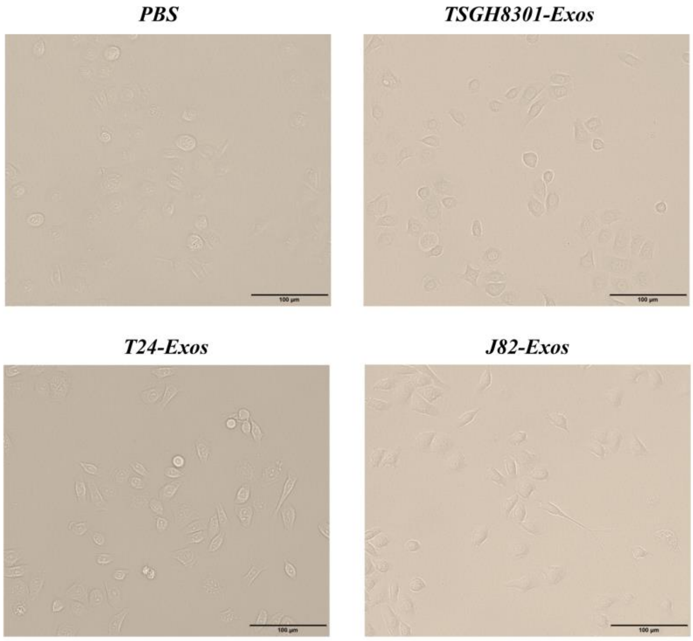

Supplementary Figure S1. The morphology of the low-grade cells gradually changes into spindle-shaped mesenchymal-like after treatment of high-grade bladder cancer cells conditioned media.

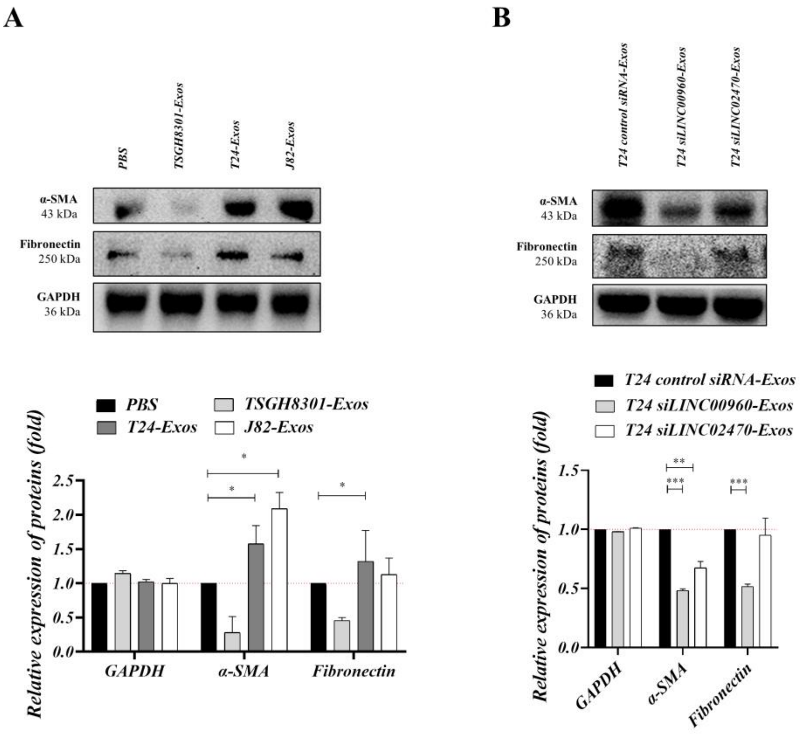

Supplementary Figure S2. (A) Exosomes mediated the expression of EMT effectors: α-SMA and Fibronectin were compared after treatment with exosomes. (B) Exosomes derived from LINC00960-knockdown or LINC02470-knockdown T24 cell reduced the expression levels of EMT effectors, α-SMA and Fibronectin, in recipient TSGH-8301 cells when compared after treatment with each exosome. The bar-charts showed the mean and SD of triplicate experiments, * $p < 0.05$, ** $p < 0.01$, *** $p < 0.001$.
